# Aquaporins in Urinary Extracellular Vesicles (Exosomes)

**DOI:** 10.3390/ijms17060957

**Published:** 2016-06-17

**Authors:** Sayaka Oshikawa, Hiroko Sonoda, Masahiro Ikeda

**Affiliations:** Department of Veterinary Pharmacology, University of Miyazaki, Miyazaki 889-2192, Japan; oshikawa.sayaka.g3@cc.miyazaki-u.ac.jp (S.O.); sonoda-h@cc.miyazaki-u.ac.jp (H.S.)

**Keywords:** exosomes, extracellular vesicles, aquaporin-1, aquaporin-2, kidney, urine

## Abstract

Since the successful characterization of urinary extracellular vesicles (uEVs) by Knepper’s group in 2004, these vesicles have been a focus of intense basic and translational research worldwide, with the aim of developing novel biomarkers and therapeutics for renal disease. Along with these studies, there is growing evidence that aquaporins (AQPs), water channel proteins, in uEVs have the potential to be diagnostically useful. In this review, we highlight current knowledge of AQPs in uEVs from their discovery to clinical application.

## 1. Introduction

Intercellular communication in animals and plants is a fundamental biological process, known to be mediated through direct contact between cells, or molecules secreted by them, including neurotransmitters, hormones, and cytokines. In addition to these channels of communication, extracellular vesicles (EVs) including exosomes have recently become a focus of interest in biology and medicine as intercellular communication tools [1,2]. Exosomes were originally thought to be “garbage bag” organelles for disposal of unnecessary proteins and lipids into the extracellular environment [1,2,3,4,5]. However, since the discovery of exosome-mediated transfer of mRNAs and microRNAs, EVs have also been recognized as communication mediators [6,7,8], and accumulated evidence now suggests that they might have potential diagnostic and therapeutic applications in various medicinal fields [1,2].

EVs are classified into several subsets, including exosomes, microvesicles, and apoptotic bodies, all differing in both size and biogenesis [1,2,9]. Microvesicles, 50–2000 nm in diameter, bud directly from the cell surface, whereas apoptotic bodies, 500–4000 nm in diameter, are generated during the process of apoptosis. In contrast to these EVs, exosomes, which are 30–150 nm in diameter, are derived from so-called multivesicular endosomes or multivesicular bodies (MVBs). Unfortunately, the material isolated using any of the currently available methods, including differential centrifugation, polymer-based precipitation, or immunocapture by antibody-coated beads, is known to contain a mixture of EVs, which precludes clear identification of the function of each EV subset [1]. In this review, we use the terms employed in previous papers to describe the various EVs.

## 2. Biogenesis of Exosomes

This review provides an outline of the aquaporins (AQPs) in urinary EVs. Several previous studies have indicated that AQPs exist in urinary exosomes [10,11,12,13]. Therefore, here we briefly describe the process of exosome generation.

**Figure 1:**
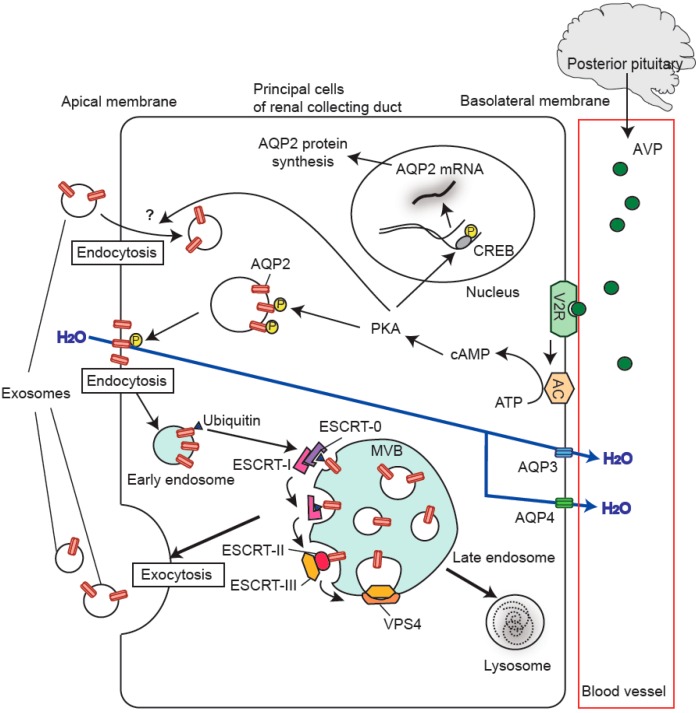
An illustration of the genesis of exosomal aquaporin-2 (AQP2) in renal collecting duct principal cells. Activation of the V2 receptor (V2R) by vasopressin (AVP) released from the posterior pituitary stimulates adenylate cyclase (AC), resulting in an increase of cAMP, thereby activating protein kinase A (PKA). This protein kinase phosphorylates AQP2, thus increasing the expression of AQP2 in the apical membrane through a vesicular trafficking mechanism. The PKA also phosphorylates CRE binding protein (CREB), and this phosphorylated protein enhances transcription of the AQP2 gene, leading to an increase in the synthesis of AQP2 protein. When apical AQP2 is ubiquitinated, the ubiquitinated AQP2 is endocytosed. Endosomal sorting complex required for transport (ESCRT)-0 then captures the ubiquitinated AQP2, and this interaction activates the sequential reactions mediated by ESCRT-I, ESCRT-II, and ESCRT-III. Finally, the endosomal membrane is excised from the endosome membrane through the action of ESCRT III and vacuolar protein sorting 4 (VPS4), thus generating multivesicular bodies (MVBs) including intraluminal vesicles. Once the MVBs fuse with the plasma membrane, the intraluminal vesicles are released as exosomes into the extracellular space. A recent paper has also suggested that an endocytotic pathway for exosomes is stimulated by activation of the V2 receptor in renal collecting duct principal cells [16].

In 1983, release of intralumical vesicles (IVs) from MVBs in reticulocytes was observed for the first time, and, in 1987, the released vesicles were named exosomes [3,4,5]. The best described mechanism for biogenesis of MVBs and exosomes is that mediated by the endosomal sorting complex required for transport (ESCRT) machinery, which is made up of the components ESCRT-0, -I, -II, and -III (Figure 1) [1,2,14]. The ESCRT machinery, together with ESCRT-associated proteins (VPS4, ALIX, *etc.*) and mono-ubiquitination of cargo, are required for MVB formation. The initial step is the recognition and recruitment of ubiquitinated cargo proteins (e.g., transmembrane proteins) by ESCRT-0 at the endosome membrane, and invagination of the endosome membrane is thereafter initiated. A component of ESCRT-0, hepatocyte growth factor-regulated tyrosine kinase substrate (HRS), recruits tumor susceptibility gene 101 (TSG101), a component of ESCRT-I, and ESCRT-I then facilitates the recruitment of ESCRT-III through the involvement of ESCRT-II and apoptosis-linked gene 2-interacting protein X (ALIX). ESCRT-III drives invagination and membrane fission associated with vacuolar protein sorting 4 (VPS4). As a result, IVs are formed inside the MVBs. When MVBs are conveyed along the secretion pathway, the IVs are finally released into the extracellular milieu as exosomes. So far, TSG101 and ALIX have been utilized as exosomal marker proteins in urine [15].

Although clearer identification of the signaling cascade is required, some studies have also shown that exosomes are formed through ESCRT-independent mechanisms [17], including the pathways mediated by tetraspanin CD63 [18], neutral sphingomyelinase [19], and ADP ribosylation factor 6 and its effector phospholipase, D2 [20].

## 3. Aquaporins in the Kidney

AQPs, water channel proteins, are a family of transmembrane proteins. Since the isolation of AQP1 from human erythrocytes by Agre’s group in 1992 [21], thirteen aquaporins (AQP0–12) have been identified in mammals [22,23]. Among them, at least eight aquaporins (AQP1–4, 6–8, 11) have been found in renal epithelial cells, which are the major source of urinary EVs [24].

AQP1, initially named CHIP28 (channel-forming integral membrane protein of 28 kDa) [21], is an archetypal molecular water channel expressed at both the apical and basolateral membranes of the proximal tubule [25], where a large amount of water is reabsorbed. AQP1 is also localized in both the thin descending loop of Henle and the descending vasa recta, which are known to be important regions for maintaining the corticomedullary osmotic gradient in the kidney [26,27]. Studies of AQP1-deficient humans and AQP1-knockout mice have highlighted the importance of AQP1 in the urinary concentration mechanism. In two unrelated individuals lacking AQP1, after either water deprivation or treatment with desmopressin, an analog of vasopressin (antidiuretic hormone), urinary osmolality was not increased [28]. Moreover, in AQP1-knockout mice, urinary osmolality was not increased under similar conditions [29]. These data indicate that AQP1 plays an important role in the renal urinary concentration mechanism, possibly mediated by creation of a renal hypertonic medullary interstitium.

AQP2 was first described by Sasaki’s group in 1993 [30]; thereafter, it has become the most extensively studied AQP protein because of its regulation by vasopressin [23]. Vasopressin, secreted from the posterior pituitary in response to an increase in plasma osmolality, hypovolemia/hypotension, or both, binds to and activates the V2 vasopressin receptor on the basolateral membrane of principal cells in the kidney collecting duct (Figure 1). Activation of the receptor causes a sequential intracellular signaling response, including activation of adenylate cyclase (AC), generation of cAMP, and activation of protein kinase A (PKA). The activated PKA then phosphorylates AQP2 and CRE-binding protein (CREB). After the phosphorylation of AQP2, AQP2-containing vesicles are transported to the apical membrane, causing accumulation of AQP2 in the apical membrane and thereby increasing influx of water molecules across the apical membrane of collecting duct principal cells. The phosphorylated CREB is known to stimulate transcription of the *AQP2* gene, thus contributing to an increase in the total level of renal AQP2 expression. Abnormality of the renal AQP2 level is found in several water-balance disorders, including syndrome of inappropriate secretion of antidiuretic hormone (SIADH), central diabetes insipidus (DI), and nephrogenic DI [23].

AQP3 [31] and AQP4 [32] are known to be expressed in the basolateral membranes of the collecting duct system, and provide exit pathways for water molecules that enter across the apical membrane through AQP2 toward the renal interstitium [23,24]. AQP6 [33] is present in intracellular vesicles of renal collecting duct intercalated cells and functions as a type of anion channel [34]. AQP7 [35] is expressed in the brush border of the proximal tubule, and its function is thought to be related to reabsorption of glycerol [36]. AQP11 is distributed intracellularly, probably at the endoplasmic reticulum of proximal tubule cells, where it may play a role in postnatal renal development [37].

## 4. Discovery of Aquaporins in Urinary Extracellular Vesicles

Among the AQPs, only AQP1 and AQP2 have been identified in urinary EVs, AQP2 having been first discovered by Sasaki’s group in 1995 [10]. They obtained urine from human subjects and fractionated the urine by sequential centrifugation (first at 2000 rpm for 15 min and then at 120,000× *g* for 30 min). As judged by immunoblotting, they detected AQP2 in the sediment obtained by the second centrifugation. Subsequent immunoelectron-microscopy analysis of the second sediment clearly demonstrated immunogold labeling of membrane structures forming vesicle-like shapes. Subsequently, Nielsen’s group clearly confirmed these observations with rat urine, and furthermore demonstrated that AQP1, but not AQP3, was present in urinary vesicles [11]. In 2004, Pisitkun *et al.* (Knepper’s group) performed proteomic analysis of urinary exosomes and identified 295 proteins, including AQP1 and AQP2 [12]. They also analyzed the subcellular distribution of the identified proteins and showed that 24.7% and 16.3% of them were associated with endosomal trafficking and the plasma membrane, respectively. Thereafter, Knepper’s group re-evaluated human urinary exosomes using a highly sensitive LC–MS/MS system and found 1132 proteins, including 34 that are known to be related to renal diseases [38]. The results of these proteomic analyses and many other studies of mammalian urinary EVs were assembled into a database named Vesiclepedia (available at: http://microvesicles.org) [39].

So far, we and other groups have tried to detect AQPs other than AQP1 and AQP2 such as AQP3, AQP4, and AQP11, but these AQPs have not been found in urinary EVs ([11], our unpublished observations). Although it is unclear why these AQPs have not been detected, those that are expressed in the apical membrane of epithelial cells might be taken up selectively into urinary EVs. In support of this notion, it has been reported that AQP5, expressed in the apical membrane of salivary glands, is detectable in EVs from saliva [40].

## 5. Regulation of Urinary Release of Exosomal Aquaporins

The levels of urinary exosomal AQP1 and AQP2 have been shown to correlate with their renal protein levels. Our group investigated a rat model of renal ischemia-reperfusion (I/R) injury and found a significant positive correlation between the levels of renal AQP1 and urinary exosomal AQP1 in rats at 96 h after renal I/R [41]. Similarly, when we analyzed gentamicin-induced nephrotoxicity in rats [42], the level of urinary exosomal AQP2 was significantly decreased after seven days of treatment with gentamicin, and this was accompanied by a significant decrease in its renal abundance. These data indicate that renal protein abundance is a factor determining the level of urinary exosomal AQPs. However, in some cases, the relationship might be reversed: We have also observed a significant negative correlation between the levels of renal AQP1 and urinary exosomal AQP1 in rats at 30 h after renal I/R, suggesting that urinary exosomal excretion may control conversely the abundance of renal protein [41].

Recently, it has been reported that the level of urinary exosomal AQP1 is related to its level of apical membrane expression in renal tubules [43]. When rats were treated with acetazolamide, a diuretic that inhibits carbonic anhydrase, urinary release of exosomal AQP1 was significantly augmented, and the apical membrane expression of AQP1 in the proximal tubule was increased in parallel without any alteration in the level of total renal expression.

Taking into account the effect of vasopressin on AQP2 trafficking, vasopressin has been thought to be a regulator of the AQP2 level in urinary EVs [44]. The regulation was first reported by Wen *et al.* [11] using a urinary EV-rich fraction. They observed that, while 8-h excretion of AQP2 in urinary EVs in control rats was 0.9% of total kidney expression, treatment with desmopressin caused a three- to four-fold increase in excretion of AQP2 in urinary EVs. Dear’s group has studied regulation of the exosomal AQP2 level by vasopressin *in vitro* [45]. Employing a kidney collecting duct cell line (mCCDc11), they measured the level of AQP2 in exosomes secreted into the culture medium and found that vasopressin induced an increase in the exosomal release of AQP2. Interestingly, in that study, they also found that exosomes derived from vasopressin-stimulated cells were able to transfer the functional AQP2 to host cells. Vasopressin is well known to increase the apical membrane expression of AQP2 in renal collecting duct cells [23,44]. Although the pathway is unclear, enhanced apical membrane expression of AQP2 might increase urinary vesicular excretion of AQP2, as observed for AQP1.

Recently, a new hypothesis that vasopressin stimulates the uptake of EVs by renal epithelial cells was proposed. Oosthuyzen *et al.* [16] have found that desmopressin stimulates the uptake of EVs into cultured kidney epithelial cells. Through pharmacological studies, they have also found that the pathway for uptake of EVs by renal cells in response to desmopressin includes V2 receptor activation, cAMP production, PKA activation, and dynamin activation (endocytotic pathway). These observations suggest that vasopressin might increase both the uptake and release of EVs by collecting duct cells.

Our group has reported that treatment with NaHCO_3_, a urinary alkalizing agent, increases the urinary exosomal release of AQP2, and that this increase is partially but not completely inhibited by coadministration of a V2 receptor antagonist [46]. Although further clarification is required, these data suggest that urine alkalinization might be an independent regulatory factor for exosomal release of AQP2.

## 6. Aquaporins in Urinary Extracellular Vesicles as Potential Biomarkers of Renal Disease

It has been reported that AQPs in urinary EVs can be potential biomarkers for various kidney diseases (Table 1).

Acute kidney injury (AKI) is a common clinical syndrome resulting from a rapid decline in glomerular filtration rate, and the mortality and morbidity associated with AKI continue to be alarmingly high. One of the major reasons for this is the lack of an appropriate and early diagnostic marker for AKI, which is often caused by I/R injury. Sonoda *et al.* [41] have reported that a decrease in urinary exosomal AQP1 allowed early to late detection of renal I/R injury in a rat model. They also observed a similar decrease of urinary exosomal AQP1 in a recipient patient 48 h after renal allograft transplantation, as renal I/R is inevitable during this procedure.

Currently available clinical tests are not adequate for predicting the course of urinary tract obstruction. Li *et al.* [47] have reported that AQP1 in urinary EVs could be a possible candidate marker. They observed that urinary exosomal release of AQP1 was reduced in patients with unilateral pelviureteral junction obstruction who underwent pyeloplasty.

It should be noted that a series of studies by Morrissey’s group has shown that urinary AQP1 could be a possibly sensitive and specific biomarker of renal tumors, such as the clear cell or papillary subtypes of kidney cancer [49,50]. However, they only measured AQP1 in whole urine, and future studies therefore will need to determine whether similar results would be obtained using the urinary fraction of EVs.

AQP2 in urine is reportedly a good marker for the effect of vasopressin on the renal collecting ducts, and many studies have measured the level of AQP2 in whole urine of patients with various diseases related to imbalance of water homeostasis, such as hypertension, DI, SIADH, heart failure, liver cirrhosis, and pregnancy. The collected data have indicated (1) an increase of urinary AQP2 in patients with essential hypertension receiving saline infusion [51], congestive heart failure [52], SIADH [53], liver cirrhosis [54], and pregnancy [55]; and (2) a decrease of urinary AQP2 in patients with DI [10,56]. Since urinary AQP2 has been reportedly detected in the vesicular fraction but not in the soluble fraction [11,13], AQP2 in uEVs is a strong candidate for detection of water balance disorders. Supportingly, de Oliveira *et al.* [48] have shown that a reduction in the level of urinary exosomal AQP2 can be a useful biomarker of urinary concentrating defects in patients with American cutaneous leishmaniasis.

As well as disorders of water balance, we have observed that urinary exosomal AQP2 is useful for detection of renal injury caused by gentamicin [42]. Although gentamicin is widely used for treatment of many types of bacterial infection, its clinical usage has some limitations because of side effects such as nephrotoxicity and ototoxicity. In order to find a new biomarker that can be used to detect gentamicin-induced renal injury, we examined urinary exosomal proteins in rats treated with gentamicin. Short-term treatment with gentamicin (within two days) increased the urinary excretion of exosomal AQP2 and a marker protein of urinary exosomes, TSG101. These results indicate that urinary AQP2 is able to detect early renal tubule injury due to gentamicin, and the underlying mechanism may involve release of a greater number of exosomes into the urine. On the other hand, chronic treatment with gentamicin (7 days) markedly decreased the excretion of exosomal AQP2 but not that of exosomal TSG101. At this time point, the renal expression level of AQP2 was decreased. These observations suggest that exosomal AQP2 can also be used to probe late gentamicin-induced nephrotoxicity that is accompanied by a decrease in its renal protein level.

## 7. Conclusions

The discovery of AQPs has dramatically improved our understanding of water homeostasis in living organisms. Functional identification of EVs, especially exosomes, has opened a new avenue in diagnostics and therapeutics of renal diseases, and the link between AQPs and EVs has important implications for the development of novel biomarkers.

## Figures and Tables

**Table 1:** Urinary exosomal aquaporins (AQPs) related to kidney disease.

AQPs	Disease	Species	Results	Refs
AQP1	Ischemia-Reperfusion (I/R) injury	rat	↓	[41]
	Renal transplantation	human	↓	[41]
	Urinary tract obstruction	human	↓	[47]
AQP2	Urinary concentration defects	human	↓	[48]
	Gentamicin-induced nephrotoxicity	rat	↓	[42]

↓, decreased.

## Abbreviations

AC	adenylate cyclase
AKI	acute kidney injury
ALIX	apoptosis-linked gene 2-interacting protein X
AQP	aquaporin
CREB	CRE-binding protein
DI	diabetes insipidus
ESCRT	endosomal sorting complex required for transport
EV	extracellular vesicle
HRS	hepatocyte growth factor-regulated tyrosine kinase substrate
I/R	ischemia-reperfusion
IV	intralumical vesicle
MVB	multivesicular body
PKA	protein kinase A
SIADH	syndrome of inappropriate secretion of antidiuretic hormone
TSG101	tumor susceptibility gene 101
VPS4	vacuolar protein sorting 4
